# The Moral Sense

**Published:** 1915-03

**Authors:** 


### The Moral Sense.

We have just seen how man is developed from both organic and social heredity, and how the animal, only from organic heredity.

The moral sense in man, as well as his conscience, is both innate and social, and this ethical instinct is the determining factor in his social evolution. It varies greatly in degree with each individual, and while innate, it is more largely developed by social heredity. Its fineness of quality will depend upon the education and environments influencing its development.

On this moral sense, as a basis, the moral code of any generation or civilization is founded through its social heredity. Not being organic, it is, therefore, purely artificial and must be acquired by each successive generation. This accounts for the difference of morals found to obtain with every country or race. It also explains the dilemma many writers fall into in discussing sex morality. On this question social heredity and organic heredity are not distinguished between and consequently confusion arises as to what attributes are primal and which are acquired.

For example, considered from the standpoint of organic heredity it would be perfectly logical to regard the sex function as an end solely to procreation; and being natural, it could never be immoral, regardless of social conventionalities. But this overlooks the other equally true factor of social heredity, which plays even a more important part in the development of the social character. If society, for instance, has found that monogamy and moral rectiture is best for the race and that certain personal relations are immoral and injurious, this mores becomes a heritable social factor, and, as such, it becomes very material. The advocates for "free love," in the adjustment of personal relations, base their contentions on this "natural" phenomenon, but overlook the ethical character as a heritable social factor.

But in this connection it would be well to point out the fact that since moral codes are so determined and influenced they undergo more rapid changes than do innate characters, and, consequently, they may become totally different in one generation from that of the next. For we who must stamp our influence on the character of the generations yet to come should hold our social instincts as binding, though what we regard as a moral standard now may be very different for the next generation or other civilizations.

